# Interaction of Nerve Growth Factor β with Adiponectin and SPARC Oppositely Modulates its Biological Activity

**DOI:** 10.3390/ijms20071541

**Published:** 2019-03-27

**Authors:** Yuu Okura, Takeshi Imao, Seisuke Murashima, Haruki Shibata, Akihiro Kamikavwa, Yuko Okamatsu-Ogura, Masayuki Saito, Kazuhiro Kimura

**Affiliations:** 1 Department of Biomedical Sciences, Graduate School of Veterinary Medicine, Hokkaido University, Sapporo 060-0818, Japan; okura.yuu@icloud.com (Y.O.); t-imao@mfour.med.kyoto-u.ac.jp (T.I.); murashima@daktari.info (S.M.); akami@obihiro.ac.jp (A.K.); y-okamatsu@vetmed.hokudai.ac.jp (Y.O.-O.); ms-consa@krf.biglobe.ne.jp (M.S.); 2 Morinaga Institute of Biological Science, Yokohama 236-0003, Japan; h.shibata@miobs.com

**Keywords:** adiponectin, AMPK, BIAcore, extracellular signal-regulated kinase (ERK), matricellular proteins, neuritogenesis, NGFβ, PC12 cells, Secreted protein, acidic and rich in cysteine (SPARC)

## Abstract

Both adiponectin and secreted protein, acidic and rich in cysteine (SPARC) inhibit platelet-derived growth factor-BB (PDGF-BB)-induced and basic fibroblast growth factor (FGF2)-induced angiogenic activities through direct and indirect interactions. Although SPARC enhances nerve growth factor (NGF)-dependent neurogenesis, the physical interaction of NGFβ with adiponectin and SPARC remains obscure. Therefore, we first examined their intermolecular interaction by surface plasmon resonance method. NGFβ bound to immobilized SPARC with the binding constant of 59.4 nM, comparable with that of PDGF-BB (24.5 nM) but far less than that of FGF2 (14.4 µM). NGFβ bound to immobilized full length adiponectin with the binding constant of 103 nM, slightly higher than those of PDGF-BB (24.3 nM) and FGF2 (80.2 nM), respectively. Treatment of PC12 cells with SPARC did not cause mitogen-activated protein kinase (MAPK) activation and neurite outgrowth. However, simultaneous addition of SPARC with NGFβ enhanced NGFβ-induced MAPK phosphorylation and neurite outgrowth. Treatment of the cells with adiponectin increased AMP-activated protein kinase (AMPK) phosphorylation but failed to induce neurite outgrowth. Simultaneous treatment with NGFβ and adiponectin significantly reduced cell size and the number of cells with neurite, even after silencing the adiponectin receptors by their siRNA. These results indicate that NGFβ directly interacts with adiponectin and SPARC, whereas these interactions oppositely regulate NGFβ functions.

## 1. Introduction

Adiponectin, a member of the C1q/tumor necrosis factor (TNF)-related proteins, is secreted exclusively by adipocytes. Circulating adiponectin exists in several homo-oligomeric forms consisting of elemental homo-trimeric subunit with a collagen-like triple-helical structure [1,2,3,4] and its levels are lower in obese subjects compared with lean subjects [5]. Subjects carrying a missense mutation in the adiponectin gene associated with hypo-adiponectinemia exhibit the phenotype of the metabolic syndrome, including insulin resistance and coronary artery disease [1,2,3,4,6]. Administration of adiponectin has been shown to be beneficial in animal models of diabetes, obesity and atherosclerosis [1,2,3,4,6]. 

The hallmark of atherosclerosis is the uncontrolled proliferation and migration of vascular smooth muscle cells, resulting in thickening of the vascular wall [7]. Physiological concentrations of adiponectin significantly suppress both the proliferation and migration of vascular smooth muscle cells induced by platelet-derived growth factor (PDGF)-BB, through direct interaction between adiponectin and PDGF-BB [8]. Moreover, it was also shown that adiponectin binds with basic fibroblast growth factor (FGF2), thereby precluding the biological activity [9]. 

Matricellular proteins are defined as extracellular matrix (ECM)-associated proteins that have no structural roles in ECM-like collagens and laminins. Secreted protein, acidic and rich in cysteine (SPARC), also known as osteonectin and BM-40, is a collagen-binding matricellular protein that regulates tissue remodeling and repair, morphogenesis and angiogenesis in vivo [10,11]. SPARC also plays pivotal roles in altering cancer cell activity and the microenvironment of tumors as well as in the pathologies of obesity and diabetes [12,13,14]. Some SPARC functions are mediated by its binding to target molecules and alterations in their biological functions. For example, like adiponectin, SPARC binds to PDGF-AB and PDGF-BB, resulting in inhibiting the ligand binding to their receptors [15]. However, SPARC influences biological activities of FGF2 not through their direct binding [10,16]. 

SPARC protein has been detected in the brain, mainly in glia and astrocytes [17]. Although no obvious neural defects were observed in SPARC null mice [18], recent findings suggest that SPARC is involved in synaptogenesis [19] and synapse elimination [20] as well as nerve growth factor (NGF)-dependent neurite outgrowth [21,22] and axon regeneration [23]. However, it remains unclear whether SPARC directly interacts with NGF. 

NGF is a member of a family of neurotrophic factors, which is responsible for the survival, development and function of basal forebrain cholinergic neuron in the central nervous system and of peripheral sympathetic and embryonic sensory neurons [24]. NGF gene is also expressed in white adipose tissues [25]. NGF expression and secretion in 3T3-L1 adipocyte culture are markedly increased in response to inflammatory cytokine such as TNF [25]. Moreover, circulating NGF levels are upregulated in a group of women with obesity and metabolic syndrome, which are related to a low-grade systemic inflammation [26,27]. As NGF modulates various immune cell functions [28,29], it is likely that NGF plays roles as an inflammatory mediator in adipose tissues, in addition to roles as a neurotrophic factor. 

It currently remains unclear whether adiponectin affect biological activity of NGF through physical interaction. Therefore, we examined the interactions of adiponectin and SPARC with NGF using a surface plasmon resonance (SPR) method and their effects on NGF-dependent morphological changes in PC12 rat pheochromocytoma.

## 2. Results

The interactions of PDGF-BB and FGF2 with SPRAC were examined using the SPR method. Infusion of different doses of two growth factors on the surface of immobilized SPARC increased RU, reflecting their binding to the ligand, while the cessation of this infusion decreased RU, reflecting their dissociation from the ligand (Figure S1). An analysis of binding kinetics revealed that K_D_ of PDGF-BB was 24.3 nM, while that of FGF2 was 14.4 µM, triple-digit difference from the former (Table 1). On the other hand, the infusion of increasing concentrations of NGFβ gave clear sensorgrams with a K_D_ of 59.4 nM, comparable with that of PDGF-BB.

Interactions of PDGF-BB and FGF2 with full length adiponectin were also examined and found the K_D_ of PDGF-BB and FGF2 were 24.5 nM and 80.2 nM, respectively (Figure S2, Table 2). Infusion of NGFβ but not the boiled protein, increased RU in a dose-dependent manner. The K_D_ of NGFβ to adiponectin was 103 nM, comparable to those of PDGF-BB and FGF2. To determine whether the interactions of growth factors with full length adiponectin occurred through its globular region, the SPR analyses with globular adiponectin-immobilized chip were performed. Both PDGF-BB and NGFβ bound selectively to the chip with K_D_ of 70.4 nM and 1260 nM, respectively (Figure S2, Table 2).

**Figure 1:**
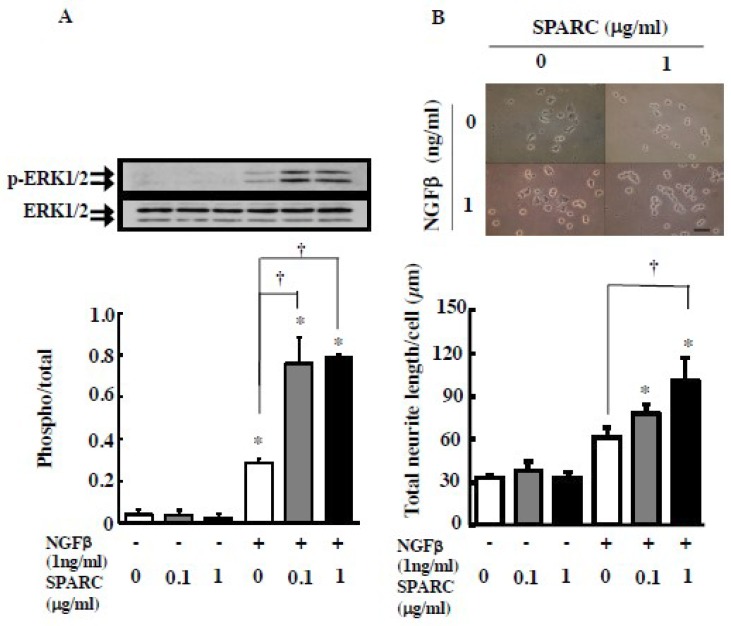
SPARC enhances NGFβ-dependent ERK activation and neurite outgrowth in PC12 cells. (**A**) PC12 cells were treated with NGFβ (1 ng/mL) in the presence or absence of SPARC (0.1 or 1 µg/mL) for 10 min. Representative results of Western blots for ERK and its phosphorylation are shown in the upper panel, Results from four independent experiments are summarized in the bottom panel. (**B**) PC12 cells were treated with or without NGFβ (0 or 1 ng/mL) either in the presence or absence of SPARC (0.1 or 1 µg/mL) for 96 h. Representative results of cells with neurites are shown in the upper panel and results (total neurite length per cell) from three independent experiments are summarized in the bottom. The length of the scale bar in the picture is 50 µm. * and † indicate significant differences (*p* < 0.05) between *no* NGFβ treatment (0 ng/mL) vs NGF treated and *no* SPARC treatment (0 µg/mL) vs SPARC treated, respectively.

In order to investigate the effects of the NGFβ and SPARC interaction on NGFβ-induced neuronal differentiation of PC12 rat pheochromocytoma, we initially examined the NGFβ-dependent activation of p44/p42 MAPK (ERK1/2) as its phosphorylated state. In the cells, among the neurotrophin receptor genes, mRNAs of TrkA, TrkC and p75NTR but not TrkB were detected (Figure S3A). Addition of NGFβ to the PC12 culture for 10 min dose-dependently induced the phosphorylation of ERK1/2, whereas neurotrophin (NT)-3 and NT4 failed to stimulate its phosphorylation (Figure S3B), suggesting that NGFβ activates the ERK signal through a TrkA neurotrophin receptor. Addition of SPARC alone did not change the phosphorylated state of ERK1/2 (Figure 1A). However, simultaneous addition of SPARC with NGFβ enhanced NGFβ-induced ERK1/2 phosphorylation. Similar to this short-term synergistic effect of SPARC and NGF, simultaneous addition of both for 96 h enhanced NGF-induced neurite outgrowth; however, SPARC alone did not influence neuritogenesis (Figure 1B).

**Figure 2:**
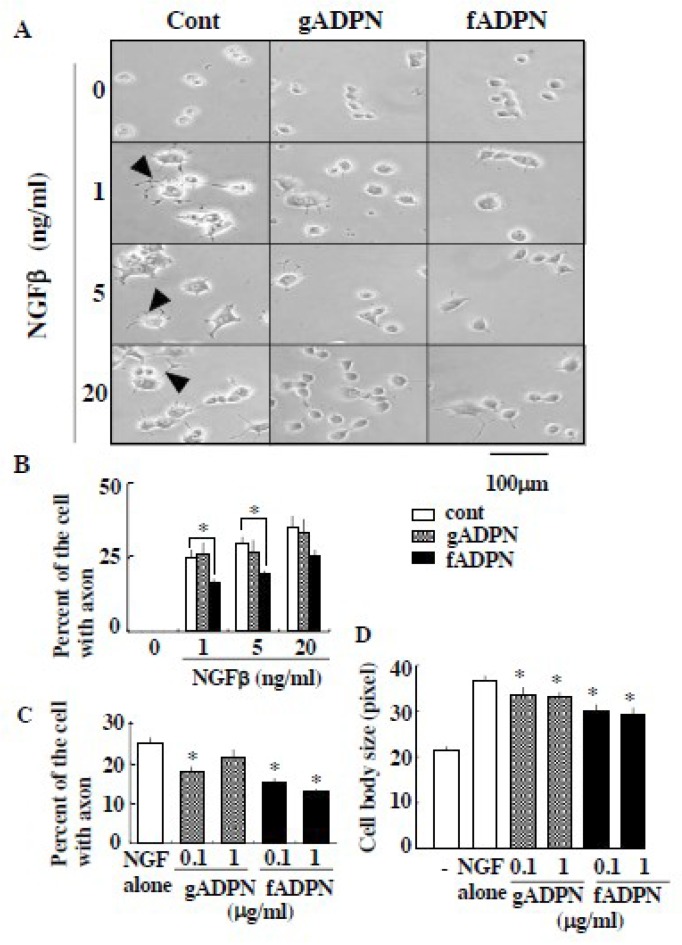
Adiponectin suppressed NGFβ-induced neurite outgrowth and cell swelling. (**A**,**B**) PC12 cells were treated with increasing concentration of NGFβ either in the presence or absence of full-length adiponectin (fADPN, 1 µg/mL) and globular adiponectin (gADPN, 1 µg/mL). Representative results of the cells (arrowhead: neurite) are shown in A and results (percentage of the cells with axon) from five independent experiments are summarized in B. (**C**,**D**) PC12 cells were treated with NGFβ (1 ng/mL) either in the presence or absence of full length adiponectin and globular adiponectin (0.1 and 1 g/mL). The ratio of the cell with axon (**C**) and the changes in cell body size (**D**) are determined and summarized from three independent experiments. * indicates the statistically significant difference (*p* < 0.05) from NGFβ treatment alone (Cont).

We next examined the interaction between NGFβ and adiponectin in the physiological condition. PC12 cells treated with NGFβ induced neurite outgrowth as well as enlargement of cell size, while the cells treated with full length adiponectin or globular adiponectin alone did not (Figure 2A). The cells treated simultaneously with NGFβ and full length adiponectin induced morphological changes of the cells but the degrees of neurite outgrowth (Figure 2B,C) and cell enlargement (Figure 2D) were significantly decreased, compared with those of low-dose NGFβ alone (e.g., 1ng/mL). It is interesting to note that full length adiponectin failed to suppress high-dose NGFβ (20 ng/mL)-induced number of cells with neurite (Figure 2A,B). Simultaneous addition of NGFβ and globular adiponectin also suppressed morphological changes of the cells in some cases but the magnitudes of suppression by globular adiponectin, if present, were less than those induced by full length adiponectin (Figure 2B–D).

**Figure 3:**
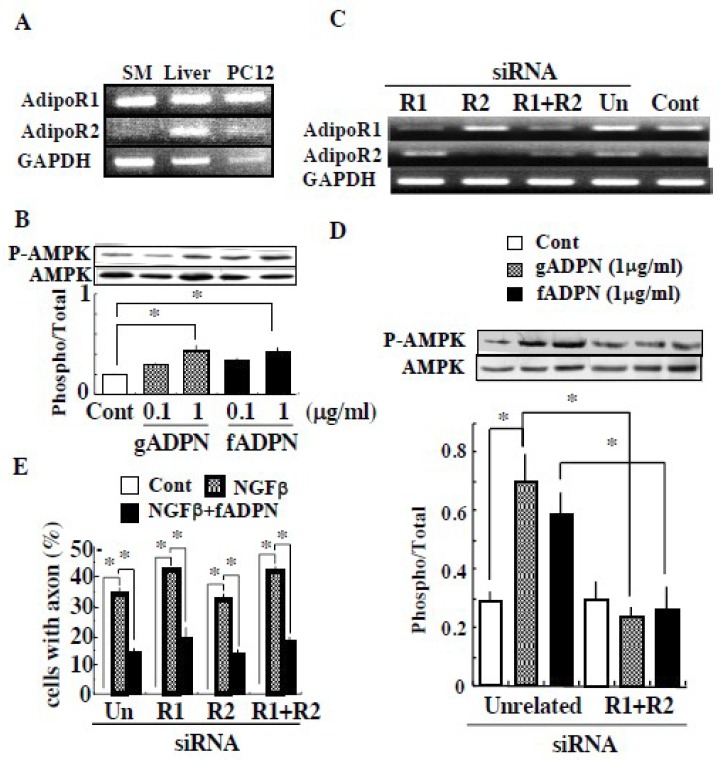
Adiponectin suppressed NGFβ-induced neurite outgrowth independently of its receptor activation. (**A**) Expression of AdipoR1 and AdipoR2 mRNA in the rat skeletal muscle (SM), liver and PC12 cells are shown. (**B**) PC12 cells were treated with full length adiponectin or globular adiponectin and the amounts of phosphorylated and total AMPK were determined. Representative results and the ratio of phosphorylated and total AMPK are shown (*n* = 5). (**C**–**E**) PC12 cells were treated with unrelated (un), AdipoR1, AdipoR2 and R1 plus R2 siRNA and (**C**) mRNA expression of AdipoR1 and AdipoR2 are shown. (**D**) The transfected cells were treated with vehicle (cont.), globular adiponectin (1 µg/mL) and full length adiponectin (1 µg/mL) and the state of AMPK activation are shown (*n* = 3). (**E**) The transfected cells were treated with vehicle (cont.), NGFβ (1 ng/mL) or NGFβ plus full length adiponectin (1 µg/mL) and the ratios of the cell with axon are shown (*n* = 3). The transfected cells treated with vehicle did not induce any neurite (axon) as shown in Figure 2A and the ratio calculated was 0 as in Figure 2B. Thus, bar for control value of each siRNA was not seen. * indicates the statistically significant difference (*p* < 0.05) from cont. or NGFβ treatment alone.

RT-PCR analysis was performed on adiponectin receptors in PC12 cells. As shown in Figure 3A, both AdipoR1 and AdipoR2 mRNA were detected. The cells treated with either full length or globular adiponectin alone did enhance the activity-related site-specific phosphorylation of AMP-activated protein kinase (AMPK) α (Figure 3B), indicating the PC12 cells expresses two types of functional adiponectin receptors. To examine whether these receptors’ activation was necessary for the adiponectin inhibition of NGFβ functions, we tested the effect of adiponectin on NGFβ-induced neurite outgrowth in the AdipoR1- and/or AdipoR2-silenced PC12 cells. Transfection of siRNA for either AdipoR1, AdipoR2 or both successfully silenced the respective receptors in mRNA levels (Figure 3C) and AMPK activation (Figure 3D). Treatment of PC12 cells with NGFβ, irrespective of silencing of either AdipoR1 or AdipoR2, induced neurite outgrowth and the addition of full length adiponectin suppressed the effect of NGFβ (Figure 3E).

## 3. Discussion

In the present study, we showed the apparent interaction of SPARC with NGFβ, PDGF-BB and FGF2 with the K_D_ of 59.4 nM, 24.3 nM and 14.4 µM, respectively. As SPARC is reported to interfere with FGF2-induced functions not through direct binding [16], the interaction between SPARC and FGF2 on the sensor chip was unexpected although its K_D_ value was a triple-digit difference from those of two other growth factors tested. Since in vitro binding studies between SPARC and FGF2 were performed by using the RIPA buffer containing various detergents during the washing procedure, this weak interaction might be masked. Similarly, no apparent binding of SPARC to transforming growth factor (TGF) β1 is reported by in vitro binding assay [30]. However, we observed weak interaction between SPARC and TGFβ1 with the K_D_ of 24.3 µM. This weak interaction might also contribute to chimeric TGF-receptor II binding to SPARC, as the binding occurred only in the presence of TGFβ1 [30]. In contrast, it is reported that SPARC prevents PDGF-induced and vascular endothelial growth factor (VEGF)-induced biological activities through their direct interactions [31]. SPR analysis revealed that SPARC interacted selectively with VEGF-165 with the K_D_ of 54.4 nM. As the K_D_ between SPARC and NGFβ is almost the same as those of PDGF-BB and VEGF-165, direct binding of NGFβ to SPARC might be able to influence NGF activity. 

Similarly, we demonstrated that NGFβ but not denatured NGFβ by boiling, bound to full length adiponectin with the K_D_ of 103 nM, while those of PDGF-BB and FGF2 were 24.5 nM and 80.2 nM, respectively. Different from SPARC, it is reported that adiponectin binds with FGF2 as well as PDGF-BB, thereby precluding their biological activity [8,9]. As the interaction between NGFβ and adiponectin shows comparative K_D_ value with those of PDGF-BB and FGF2, the interaction might also modulate NGF activity. We also showed that NGFβ bound to globular adiponectin with much greater reduction of K_D_ value (>10-fold), compared with its binding to full length adiponectin. As the K_D_ values between PDGF-BB and either adiponectin were relatively unchanged (<3-fold), it is likely that trimeric or a much higher dimensional structure of adiponectin might be necessary for NGFβ interaction. This lower ability to bind NGFβ may lead to weaker suppressive activity by globular adiponectin of NGFβ function.

NGFβ binds to two different receptors: the TrkA tyrosine kinase receptor with high affinity and the p75 NTR with low affinity [32]. In PC12 cells, NGFβ activates the ERK signal through the TrkA, that leading to neurite outgrowth [33]. In the present study, we confirmed the NGFβ activities, whereas SPARC alone did not change the phosphorylated state of ERK1/2 and subsequent neuritogenesis. However, simultaneous addition of SPARC with NGFβ enhanced NGFβ-induced ERK1/2 phosphorylation and NGFβ-induced neurite outgrowth. This synergistic effect of SPARC and NGF on neurite outgrowth was also found in superior cervical ganglion neurons and Schwann cells [21,22]. The basal forebrain cholinergic system is one of the target neuronal networks for NGF as a survival factor of cholinergic neurons [24]. NGF is also involved in nurturing the peripheral nervous system [34]. On the other hand, SPARC in the brain is suggested to facilitate cholinergic synapse formation [35], whereas other studies show that SPARC antagonizes the synaptogenesis by having, another matricellular protein [19] and triggers a cell-autonomous program of synapse elimination in cholinergic neurons [20]. Collectively, the present results suggest that a direct interaction between SPARC and NGFβ enhances the biological activity of this growth factor in the central and peripheral nervous systems. However, the mechanism by which the SPARC and NGFβ interaction enhanced NGF-signals remains obscure and further works are needed to clarify it.

PC12 cells treated with full length adiponectin alone did not induce any morphological changes, while it activated intracellular signal such as AMPK. The cells treated simultaneously with NGFβ and full length adiponectin induced neurite outgrowth and cell swelling but the degrees of the changes were significantly less than those of NGFβ alone. In addition, increasing the concentration of NGFβ prevented suppression by the constant amount of adiponectin, suggesting that at high concentrations of NGF, excess unbound NGFβ is able to induce neuritogenesis. Moreover, as this adiponectin suppression of NGFβ-dependent morphological changes were seen even after silencing adiponectin receptor signaling in PC12 cells, therefore our present results indicate that full length adiponectin interacts with NGFβ, thereby inhibits NGFβ functions, possibly through interfering its interaction with TrkA, in contrast to SPARC. 

The physiological relevance of full length adiponectin and NGF interaction remains to be elucidated. However, it is interesting to note that NGF production in adipocytes is enhanced and adiponectin production is suppressed, by inflammatory stimuli like TNF [25]. In addition, proteases secreted from activated monocytes and/or neutrophils cleave full-length adiponectin to generate globular adiponectin [36]. Supportively, blood NGF levels are upregulated in a group of women with obesity and the metabolic syndrome [26,27], while circulating adiponectin levels are lower in obese subjects [27]. Therefore, in an adipose tissue from obese subjects where inflammation develops, it is likely to occur that the amounts of NGF and full length adiponectin are increased and decreased, respectively and as a consequence dominance of NGF over adiponectin becomes clear. Furthermore, the expression of SPARC is increased in the adipose tissue of obese animals [14]. In such conditions, NGF and SPARC interaction is expected to facilitate recruitment and activation of mast cells [37], that sustain chronic low-grade inflammation within adipose tissue [38] (see also Figure S4). In an adipose tissue from lean subjects, it is plausible that locally produced NGF is associated with full length adiponectin, resulting in the masking of NGF bioactivity (Figure S4).

In summary, we showed that NGFβ interacted substantially with both SPARC and full length adiponectin and that SPARC enhanced, but adiponectin suppressed, NGFβ-dependent function in PC12 cells. Other than a neurotrophic factor, NGF plays roles in obesity-related inflammation as described above, in the proliferation and survival of various cancers [39,40] and in pain control [41]. Further works should be undertaken to investigate the involvement of NGF interactions with adiponectin and/or SPARC in each NGF-mediated process in detail.

## 4. Materials and Methods

### 4.1. Materials

Recombinant murine full-length adiponectin was purchased from Biovender Laboratory Medicine, Inc. (Bmo, Czech Republic), while recombinant murine globular adiponectin and recombinant human PDGF-BB were purchased from Wako Pure Chemical (Osaka, Japan). NGFβ from mouse submaxillary glands was bought from Alomone Labs (Jerusalem, Israel). Recombinant human basic FGF was purchased from Acris Antibodies GmbH (Hiddenhausen, Germany), while recombinant human VEGF-165 was purchased from Becton Dickison (Bedford, MA, USA). Recombinant human TGFβ1 was purchased from R&D systems (Minneapolis, MN, USA).

### 4.2. Analysis of Protein-Protein Interaction with Surface Plasmon Resonance (SPR) Method

The interactions of a growth factor with either adiponectin or SPARC were examined using the BIAcore X instrument (GE healthcare, Tokyo, Japan) and the binding kinetics were analyzed with BIAevaluation software [42,43]. Briefly, full length adiponectin, globular adiponectin or SPARC (Sangi, Tokyo, Japan) as a ligand was immobilized onto the carboxymethylated dextran surface of the CM5 sensor chip, respectively. The relative responses for the immobilized full length and globular adiponectin and SPARC were 2025, 12707 and 3574 resonance units (RU), respectively, where 1000 RU is equivalent to 1ng of protein/mm^2^. The surface of the adiponectin-chip or the SPARC-chip was perfused with HBS-EP buffer (10 mM HEPES, 150 mM NaCl and 0.005% Surfactant P20, pH 7.4) at 37 ℃ and then with increasing concentrations of a number of growth factors (as analytes) dissolved in buffer at a flow rate of 20 µL/min for 105 s. Following the addition of each analyte, dissociation was evaluated by passing the buffer alone over the chip for 120 s. If an analyte bound to a ligand, the surface showed a change in reflected light, which was directly proportional to the mass bound and measured in arbitrary RU. Based on the dissociation constant (kd) (s^−1^) and association constant (Ka) (M^−1^s^−1^) obtained, the binding constant K_D_ (M) was calculated by dividing Kd by Ka. The regeneration of the surface of the sensor chip was performed by injecting 1 M NaCl at 20 µL/min for 90 s.

### 4.3. Assay of Biological Activity of NGF

Rat pheochromocytoma, PC12 cells were cultured in RPMI1640 medium (Wako) containing 10% fetal calf serum (FCS), 10% horse serum. To examine the effects of NGF on their morphological changes (neurite outgrowth and cell swelling), the cells (5 × 10^3^ cells) were cultured on Type I-collagen-coated plates (Iwaki Techno Glass, Chiba, Japan) in RPMI1640 containing 1% FCS and 1% horse serum for 24 h and subsequently cultured with either NGFβ, adiponectin or both for 4 days. To quantify the morphological changes of PC12 cells, at least 100 randomly selected cells per experimental condition were photographed at the same scale under light transmission inverted photomicroscopy. The picture was analyzed using Adobe Photoshop and NIH Image J, a public-domain image processing and analysis program. Changes in cell body length were measured as a marker of cell swelling. Total neurite length measured and number of cells with neurite which length was longer than cell body length counted were used as an indicator of axonal elongation [44].

### 4.4. Expression of AdipoR1 and AdipoR2 in PC12 Cells and Their Silencing

Total RNA was isolated from PC12 cells and rat tissues by the guanidine-isothiocyanate method using ISOgen reagent (Takara, Tokyo, Japan) and RNA (2 µg) was used for reverse transcription. Rat AdipoR1 (GenBank accession number NM 207587), AdipoR2 (NM 001037979) and glyceraldehyde 3-phosphate dehydrogenase (GAPDH) (NM 017008) cDNA were amplified with the primer pairs as follow: AdipoR1 (201bp) Forward: 5′-TGC TTC AAG AGC ATC TTC CG-3′, Reverse: 5′-GAA TGA CAG TAG ACG GTG TG-3′ (annealing conditions 56 ℃, 30 s, 29 cycles); AdipoR2 (206bp) Forward: 5′-TCT TCT TGG GAG CCA TTC TC-3′, Reverse: 5′-GCA CAC AGA TGA CAA TCA GG-3′ (56 ℃, 30 s, 29 cycles); GAPDH (453 bp) Forward: 5′-ACC ACA GTC CAT GCC ATC AC-3′, Reverse: 5′-TCC ACC ACC CTG TTG CTG TA-3′ (62 ℃, 30 s, 27 cycles).

Expression of AdipoR1 and AdipoR2 were suppressed by treating the cells with siRNA as essentially described by Fujioka et al [45]. In brief, siRNA specific for AdipoR1 and AdipoR2 and unrelated siRNA were transfected with Lipofectamine2000 (Invitrogen, Carlsbad, CA, USA) according to the instruction provided and cultured as described above. 

### 4.5. MAP Kinase and AMP-Activated Protein Kinase Activation

PC12 cells were grown to 80% confluence in RPMI1640 containing 10% FCS and 10% horse serum and further cultured in RPMI1640 containing 1% FCS and 1% horse serum for 24 h. Subsequently the cells were treated with either vehicle, NGFβ, neurotrophin (NT)-3, NT-4 (Sigma-Aldrich, St. Louis, MO, USA), SPARC or adiponectin for 10 min. The cells were then lysed with the lysis buffer [50 mM Hepes (pH 7.5), 150 mM NaCl, 5 mM EDTA, 10 mM sodium pyrophosphate, 2 mM NaVO_3_ containing protease inhibitor mixture (Complete; Boehringer Mannheim, GmbH, Germany) and 1% (*v/v*) Nonidet P40], centrifuged at 12,000× g for 15 min at 4 °C and the supernatant was saved at –70 °C. 

Aliquot of the lysates (15 or 20 µg of protein) were separated by SDS-PAGE (10% gel) and transferred on PVDF membranes (Immobilon, Millipore, Bedford, MA, USA). The membranes were incubated first in a blocking buffer [20 mM Tris/HCl (pH 7.5), 150 mM NaCl] containing 0.1% Tween 20 and 5% (*v/v*) skimmed milk], then in the buffer containing anti-Erk1/2, anti-phosphorylated Erk1/2 (Thr202/Tyr204), anti-AMPKα or anti-phosphorylated AMPKα (Thr172) antibody (Cell Signaling Technology, Beverly, MA, USA) for 2 h. The bound antibody was detected with horseradish peroxidase-linked secondary antibodies (Zymed Laboratories, South San Francisco, CA, USA) and an enhanced chemiluminescence system (Millipore). The intensity of chemiluminescence for the corresponding proteins was analyzed by NIH Image J.

### 4.6. Statistical Analysis

Data were expressed as means ± standard errors of the mean (SEM) and analyzed by ANOVA followed by the Tukey-Kramer post-hoc test. A *p* value of less than 0.05 was considered statistically significant. 

## Figures and Tables

**Table 1:** Summary of analyte binding to SPARC.

Analytes	Association Constant (ka)	Dissociation Constant (kd)	Binding Constant (K_D_ = kd/ka)
PDGF-BB	3.71 × 10^4^	9.03 × 10^−4^	2.43 × 10^−8^
VEGF-165	7.58 × 10^4^	4.13 × 10^−3^	5.44 × 10^−8^
FGF2	1.35 × 10^2^	1.95 × 10^−3^	1.44 × 10^−5^
TGFβ1	7.90 × 10^2^	1.92 × 10^−2^	2.43 × 10^−5^
NGFβ	1.86 × 10^5^	1.10 × 10^−2^	5.94 × 10^−8^

**Table 2:** Summary of analyte binding to adiponectin.

Analytes	Association Constant (ka)	Dissociation Constant (kd)	Binding Constant (K_D_ = kd/ka)
	(ligand: full length adiponectin)		
PDGF-BB	1.15 × 10^3^	2.82 × 10^−5^	2.45 × 10^−8^
FGF2	7.16 × 10^2^	5.76 × 10^−5^	8.02 × 10^−8^
NGFβ	5.76 × 10^4^	5.97 × 10^−3^	1.03 × 10^−7^
	(ligand: globular adiponectin)		
PDGF-BB	1.05 × 10^5^	7.38 × 10^−3^	7.04 × 10^−8^
NGFβ	1.15 × 10^4^	1.45 × 10^−2^	1.26 × 10^−6^

## Supplementary Materials

Supplementary materials can be found at https://www.mdpi.com/1422-0067/20/7/1541/s1.

## Abbreviations

AMPK	AMP-activated protein kinase
ECM	extracellular matrix
ERK	extracellular signal regulated kinase
FCS	fetal calf serum
FGF	fibroblast growth factor
GAPDH	glyceraldehyde 3-phosphate dehydrogenase
MAPK	mitogen-activated protein kinase
NGF	nerve growth factor
NT	neurotrophin
PDGF	platelet-derived growth factor
RU	resonance unit
SPARC	secreted protein, acidic and rich in cysteine
SPR	surface plasmon resonance
TGF	transforming growth factor
TNF	tumor necrosis factor
VEGF	vascular endothelial growth factor
