# Supplementary material for: Interaction of Nerve Growth Factor β with Adiponectin and SPARC Oppositely Modulates its Biological Activity
Source: Int J Mol Sci. 2019 Mar 27;20(7):1541. doi: 10.3390/ijms20071541 (PMC6479836; doi:10.3390/ijms20071541)
Supplement: Supplementary file 1 [file ijms-20-01541-s001.zip › ijms-466226-SI.pdf]

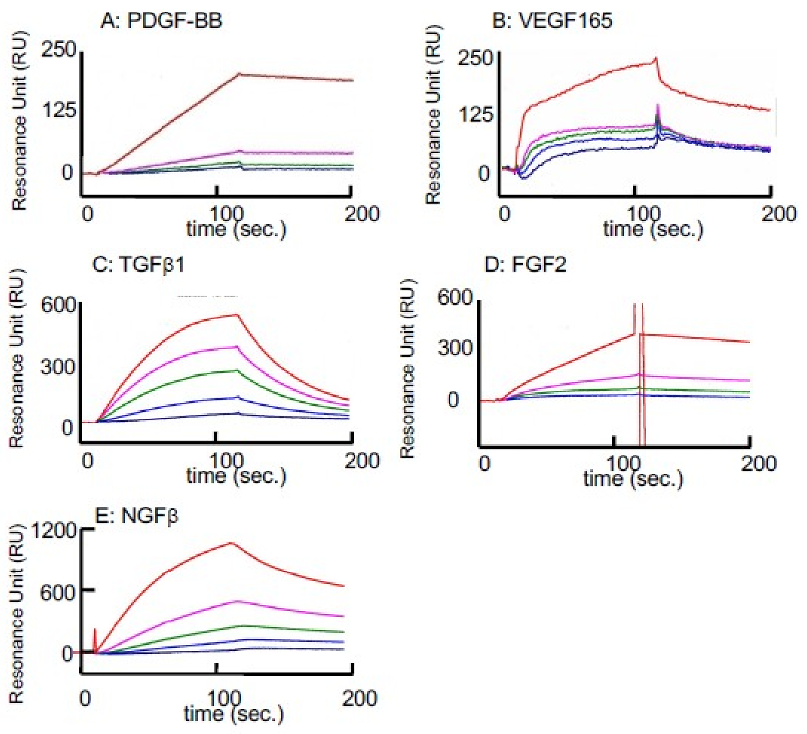

Figure 1. Binding of five growth factors to immobilized SPARC. Increasing concentrations of PDGF-BB (0.01225, 0.025, 0.05, and 0.25 μg/ml from the bottom), VEGF165 (0.25, 0.5, 0.75, 1, and 2.5 μg/ml from the bottom), TGFβ1 (0.1, 0.25, 0.5, 0.75, and 1 μg/ml from the bottom), FGF2 (0.25, 0.5, 1, and 2.5 μg/ml from the bottom), and NGFβ (0.1, 0.25, 0.5, 1, and 2.5 μg/ml from the bottom) were perfused on the surface of a SPARC-immobilized BIAcore sensor chip. Respective overlay plots of sensorgrams are shown.

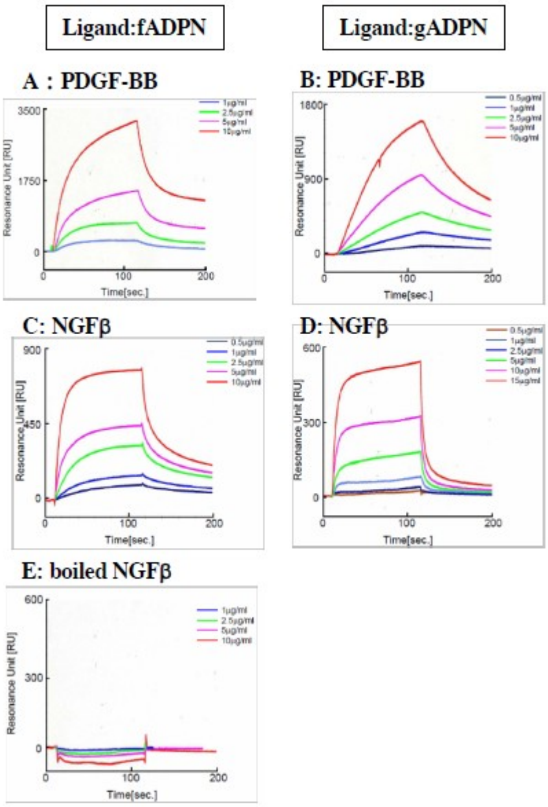

Figure 2. PDGF-BB and NGF binding to immobilized adiponectin. Increasing concentrations of PDGF-BB (A: 1, 2.5, 5, 10 μg/ml from the bottom for full length adiponectin; B: 0.5, 1, 2.5, 5, 10 μg/ml from the bottom for globular adiponectin), NGFβ (C : 0.5, 1, 2.5, 5, 10 μg/ml from the bottom for full length adiponectin; D: 0.5, 1, 2.5, 5, 10, 15 μg/ml from the bottom for globular adiponectin) and NGFβ boiled for 30 min (E : 1, 2.5, 5, 10 μg/ml from the bottom for full length adiponectin) were perfused on the surface of full-length adiponectin (fAPDN) or globular adiponectin (gADPN) immobilized BIAcore sensor chip. Respective overlay plots of sensorgram are shown.

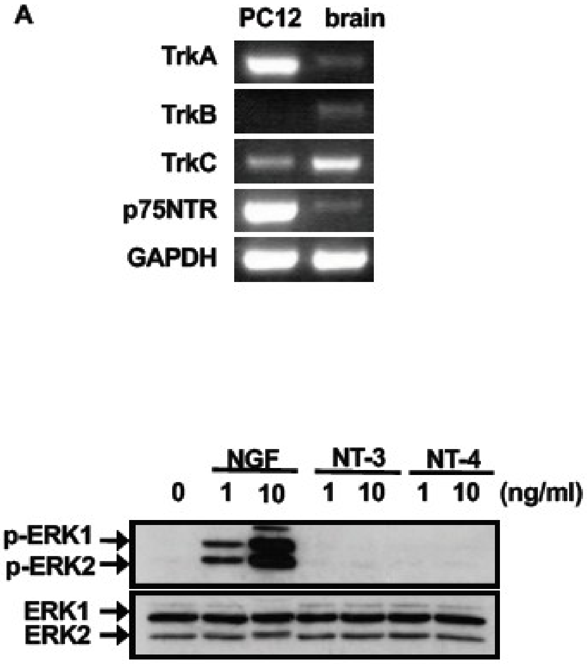

Figure 3. Expression of neurotrophin receptor mRNAs and their signals in PC12 cells. (A) The mRNA expression of neurotrophin receptor genes in PC12 cells was analyzed by RT-PCR. (B) PC12

cells were treated with NGFβ (1 and 10 ng/ml), NT3 (1 and 10 ng/ml), and NT4 (1 and 10 ng/ml) for 10 min. Representative results of Western blots for ERK and its phosphorylated form are shown.

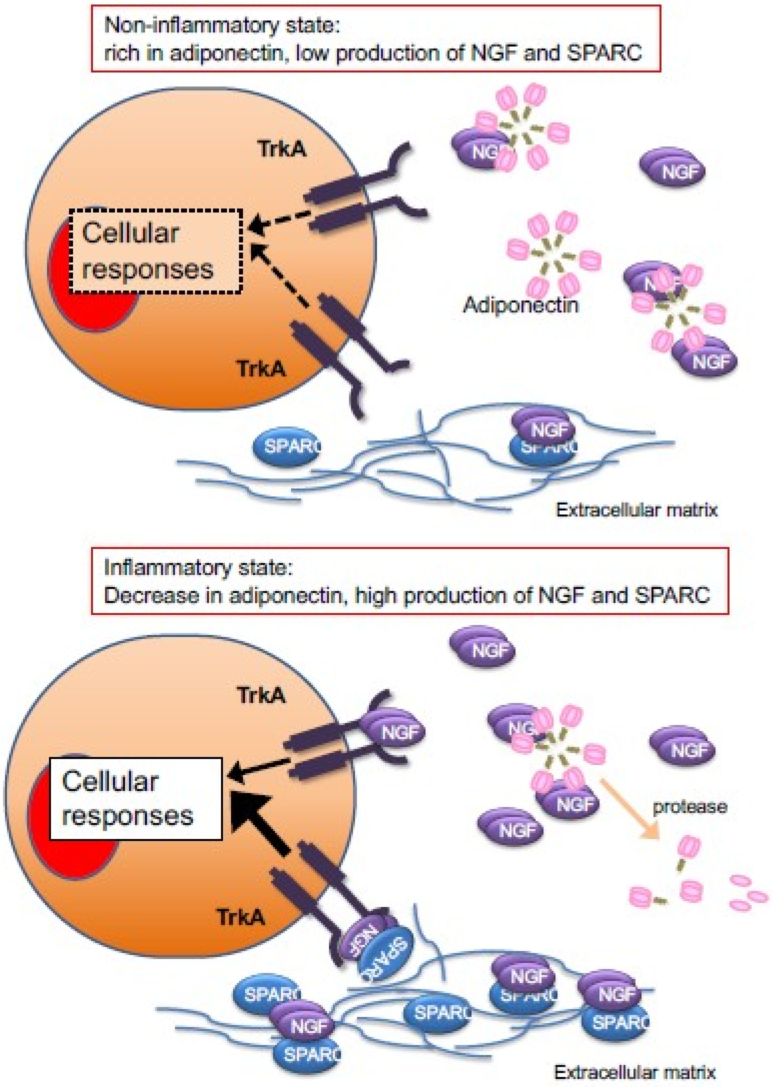

Figure 4. Possible roles of NGF interaction with adiponectin and SPARC in inflammatory and non-inflammatory conditions. As described in the discussion, supposing that the interactions occurred in the adipose tissue of obese individual, relative abundance of full length adiponectin declines due to decreased its expression and increased its degradation by proteases while both NGF and SPARC are highly induced. Therefore, NGF and SPARC interaction may potentiate NGF signals not only in a neural cell but also the other target cell such as mast cell. In contrast, in non-inflammatory state, full length adiponectin may be present sufficiently to mask basally produced NGF bioactivity.
